# International recommendations on epinephrine auto-injector doses often differ from standard weight-based guidance: a review and clinical proposals

**DOI:** 10.1186/s13223-022-00736-5

**Published:** 2022-12-05

**Authors:** Sten Dreborg, Graham Walter, Harold Kim

**Affiliations:** 1 Department of Child and Adolescent Allergology, Women’s and Children’s Health, University of Uppsala, Uppsala, Sweden; 2 Department of Medicine, Western University, London, ON Canada; 3 Division of Clinical Immunology and Allergy, Western University, London, ON Canada; 4 Department of Medicine, McMaster University, Hamilton, ON Canada

**Keywords:** Epinephrine, Epinephrine, Auto-injector, Intramuscular, Subcutaneous, Dose, LD_50_, Weight, Age

## Abstract

**Background:**

In anaphylaxis, the dosing of injectable epinephrine in medical settings has been arbitrarily recommended to be 0.01 mg/kg of body weight. For ethical reasons, there have been no dose–response studies or double-blind studies performed on patients with active anaphylaxis. Intramuscular delivery of epinephrine has been the standard. Auto-injectors for use in the treatment of anaphylaxis are available in four strengths (0.1, 0.15, 0.3, and 0.5 mg). However, in many countries, only the 0.15 and 0.3 mg strengths are available. Consequently, many adult, heavy patients are prescribed the 0.3 mg dose, which may result in only one-fifth to one-third of the recommended weight-based dose being administered in heavy patients experiencing anaphylaxis. Underdosing may have therefore contributed to mortality in anaphylaxis.

**Objective:**

To review the doses of epinephrine recommended for the treatment of anaphylaxis in the community, and assess whether recommendations should be made to increase dosing for heavy adult patients in hopes of avoiding future deaths from anaphylaxis.

**Methods:**

We reviewed multiple national and international recommendations for the dosing of epinephrine. We also reviewed the literature on adverse drug reactions from epinephrine, lethal doses of epinephrine, and epinephrine dose-finding studies.

**Results:**

The majority of national and regional professional societies and authorities recommend epinephrine delivered by auto-injectors at doses far lower than the generally accepted therapeutic dose of 0.01 mg/kg body weight. Furthermore, we found that the recommendations vary even within regions themselves.

**Conclusions:**

We suggest prescribing more appropriate doses of epinephrine auto-injectors based on weight-based recommendations. There may be some exceptions, such as for patients with heart disease. We hypothesize that these recommendations will lead to improved outcomes of anaphylaxis.

## Introduction

Epinephrine was first synthesized in 1904 independently by Friedrich Stolz [1] and Henry Drysdale Dakin [2]. Clinically, epinephrine is known to increase patient blood pressure, heart rate, and stroke volume, and have potent anti-asthmatic and anti-allergic properties. Thus, epinephrine has been the drug of choice for the treatment of severe allergic reactions for more than a century.

Within healthcare settings, epinephrine drawn from an ampoule and injected with a needle and syringe has been recommended for the treatment of systemic allergic reactions/anaphylaxis within health-care settings, using the internationally accepted (but not properly studied) dose of 0.01 mg/kg body weight. Historically, prefilled syringes were used for the treatment of anaphylaxis in the community. From the introduction of epinephrine auto-injectors in 1979 [3], the increased uptake of this delivery method has allowed caregivers, partners, and patients themselves to treat systemic allergic reactions. However, for economic reasons, epinephrine auto-injectors have not been used in most regions of the world, and have sometimes been replaced by prefilled syringes and needles.

Even when patients have access to epinephrine auto-injector(s) during reactions in the community, there have been documented cases of fatal anaphylaxis. Some possible reasons include prescription for an auto-injector with too low a dose or with a too short needle for skin to muscle distance, patients not using their auto-injector due to fear of the local pain/discomfort of injections, patients not possessing a second auto-injector on-hand when the first injection does not improve symptoms, anaphylaxis occurring in a remote area where supportive medical assistance is not within reasonable distance, and injection being performed too late in relation to the onset of symptoms.

In this review, we will present the available data on the dose of epinephrine injected by commercially available auto-injectors with fixed epinephrine content. To avoid further deaths from anaphylaxis in overweight or obese adults, we will justify a proposal for higher dosing of epinephrine auto-injectors, particularly as it pertains to adult women with higher body mass indices.

## Auto-injector doses recommended by professional societies and authorities

There are several dose recommendations for epinephrine auto-injectors as agreed upon by allergy societies and/or authorities. These doses are typically based upon the recommended in-hospital dosing of 0.01 mg (mg) epinephrine per kilogram (kg) body weight for the treatment of systemic allergic reactions; an amount which has yet to be studied scientifically. As demonstrated in Table 1, medication delivered by each fixed dose auto-injector often differs greatly from the 0.01 mg/kg recommendation. Importantly, most jurisdictions have only two or three fixed doses of epinephrine available as auto-injectors.

**Table 1:**
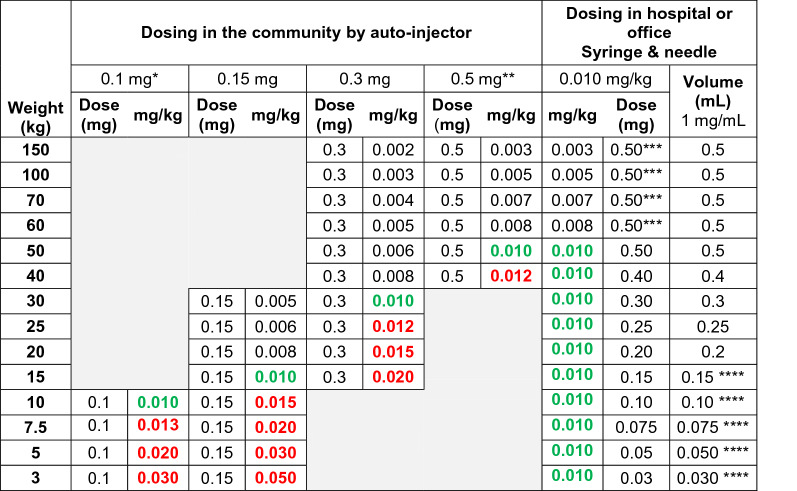
Effective epinephrine delivery per kilogram body weight by available auto-injector dose in children and adults

Colour code: Numbers in red denote doses higher than the “recommended dose” 0.01 mg/kg body weight, green color indicates “the recommended dose”, 0.01 mg/kg body weight, and black color doses lower than the “recommended” dose. Yellow areas indicate that the dose can only be prescribed in some countries, leading to (probably) under-medication

*mg* milligram, *kg* kilogram, *mL* millilitre

^*^Available only in the USA

^**^Available only in Europe and Canada

^***^IM Epinephrine administered in hospital or office typically does not exceed 0.5 mg per administration

^****^Preferably the stock solution for IV injection, 0.1 mg/mL, 1.5 mL to 0.3 mL

In conducting this review, we noted the recommendations by the World Allergy Organization (WAO), the European Academy of Allergy and Clinical Immunology (EAACI), the Australian Society of Clinical Immunology and Allergy (ASCIA), the Canadian Society of Allergy and Clinical Immunology (CSACI), the American Academy of Asthma, Allergy, and Immunology (AAAAI), the American College of Allergy, Asthma, and Immunology (ACAAI), and then the recommendations by member states.

To corroborate this data, we asked the allergy societies and allergists of several countries which recommendations are used locally. In some cases, we had to ask several individuals for information on these doses. In a small number of cases, we failed to obtain adequate information for our analysis. The recommendations by member states of EAACI do not always follow the mutual agreement published by EAACI and the recommendations vary substantially between continents. Recommendations based on body weight are illustrated in Table 2 and those based on age in Table 3.

**Table 2:**
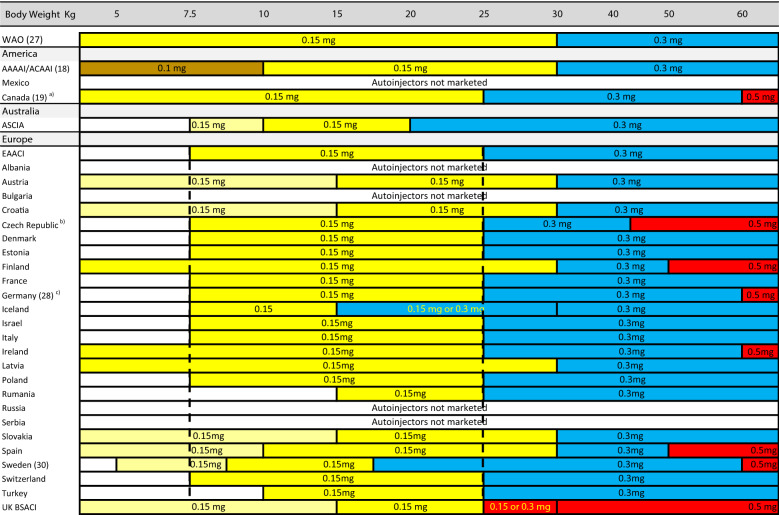
Doses of adrenaline in mg, based on body weight in kilogram, recommended by national authorities and or scientific societies

Brown colour indicates 0.1 mg adrenaline per autoinjector. Yellow colour indicates 0.15 mg adrenaline per autoinjector. Light yellow colour indicates that the dose can be prescribed by allergy specialists. Yellow text within blue or red colour indicates that either dose can be used. Blue colour indicates 0.3 mg adrenaline per autoinjector. Red colour indicates 0.5 mg adrenaline per autoinjector

Data collected during 2020 and 2021 from publications, scientific societies and or personal contacts. The vertical, black dotted lines indicate the Pan-European recommended weight for the use of 0.15 mg adrenaline. Turkey and Israel are not European countries geographically, however, members of the EAACI. (References used in Table 2, [26–29]).

*WAO* World Allergy Organization, *AAAAI*, American Academy of Asthma, Allergy and Immunology, *ACAAI* American College of Allergy, Asthma, and Immunology, *EAACI* European Axademy of Allergy and Clinical Immunology, *ASCIA* Australasian Society of Clinical Immunology and Allergy

**Table 3:**
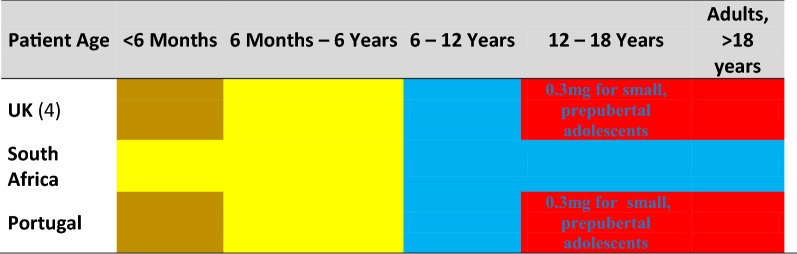
Epinephrine dosing in milligrams based on age in the UK, South Africa, and Portugal

Brown = 0.1 mg, Yellow = 0.15 mg, Blue = 0.3 mg, Red = 0.5 mg epinephrine auto-injector recommended by guidelines

**Fig. 1:**
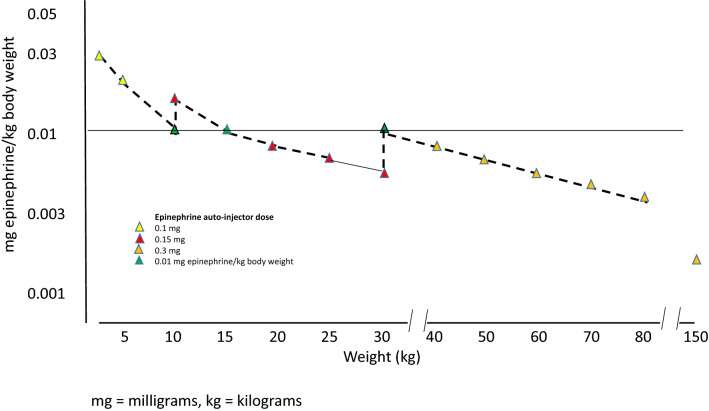
Effective epinephrine delivery per kilogram body weight by available auto-injector dose in the USA

**Fig. 2:**
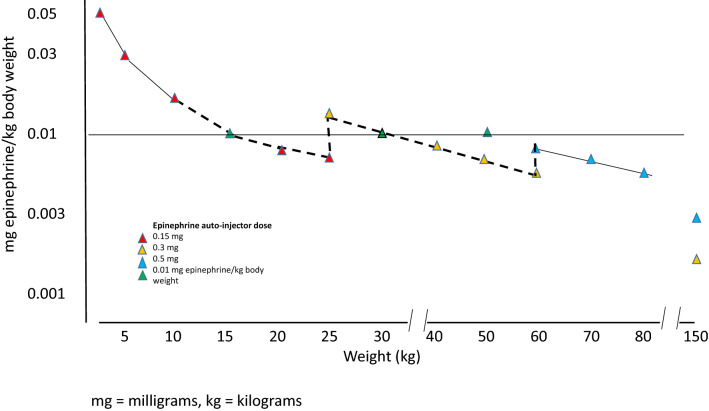
Effective epinephrine delivery per kilogram body weight by available auto-injector dose in Europe and Canada

Following these auto-injector recommendations, Fig. 1 illustrates the doses available for the treatment of anaphylaxis in the USA in relation to weight, as delivered by the three fixed doses: 0.1, 0.15, and 0.3 mg epinephrine. Figure 2 shows the same for the three concentrations available in some European countries and Canada: 0.15, 0.3, and 0.5 mg epinephrine per auto-injector. Based on these analyses, in the USA, there is a clear risk of underdosing epinephrine in any adult weighing > 30 kg. Conversely, in Europe and Canada, there is also a risk of high doses being injected in the pediatric population as well as adults weighing < 50 kg. It thus stands to reason that very few patients will get the exact 0.01 mg/kg weight-based dosing when using epinephrine auto-injectors. (Tables 1, 2, and 3, and Figs. 1 and 2).

In this review, we will examine different characteristics of epinephrine that may justify modification of the above recommendations.

## Properties and dosing of epinephrine

### Adverse drug reactions (ADRs) from epinephrine at recommended doses

In healthy patients, epinephrine may induce dizziness, restlessness, anxiety, as well as increased heart rate, increased blood pressure, and stroke volume [4–6]. When epinephrine is injected into a patient with ongoing systemic allergic reaction/anaphylaxis, the effect of epinephrine counteracts and balances many of the deleterious signs and symptoms of anaphylaxis [6].

Jajamali et al. [4] reported on a young healthy adult with no significant risk factors for coronary artery disease who developed myocardial infarction following intramuscular (IM) administration of 0.5 mg of epinephrine for an anaphylactic reaction. They conclude that the postulated mechanism was α-receptor mediated coronary vasospasm. However, the authors state that the use of epinephrine in the setting of life-threatening anaphylaxis is life-saving and the benefits far outweigh the risk of adverse effects. Further to this, coronary vasospasm anaphylaxis has been documented secondary to anaphylaxis itself; a phenomenon known as Kounis Syndrome [4].

Ruiz-Garcia et al. [5] reported on cardiovascular changes during anaphylaxis in adults undergoing peanut oral challenges and Turner et al. [6] reported on limited cardiovascular changes in response to intramuscular epinephrine after peanut oral challenge. Interestingly, the stroke volume on bedside monitoring as reported by Turner and colleagues did not reach the pre-challenge level after injection of epinephrine. However, heart rate did increase, which resulted in higher cardiac output than pre-challenge; i.e., balancing the changes resulting from systemic allergic reactions to food. These changes were not regarded as adverse drug reactions (ADR), and they demonstrated clinically acceptable effects.

The German/European [7] registry of anaphylaxis, and the European Medical Agency (EMA), were also assessed for ADRs induced by epinephrine administered during anaphylaxis. There were no severe cases of ADRs reported. This suggests that the doses that are used in practice are not at significant risk of causing any serious ADRs. Still, there may be an increased risk of ADRs in patients with known cardiac arrhythmia or other heart diseases.

In Western Europe and Canada, a 0.5 mg auto-injector (Emerade^™^ 0.5 mg) is available. There have not been any reported adverse effects of 0.5 mg epinephrine used for the treatment of general allergic reactions/anaphylaxis [8]. Further to this, there has been a recent study supporting the use of epinephrine 0.5 mg IM for patients with allergic reactions to subcutaneous allergen immunotherapy [9].

No published data support the claim or belief that children tolerate epinephrine better than adults. This idea may stem from an increased fear surrounding epinephrine use in adults with underlying cardiac disease. It stands to reason that this may be why some jurisdictions recommend lower mg per kg doses by auto-injector for adults at risk of anaphylaxis when compared to children.

### Dose finding studies

For ethical reasons, it is nearly impossible to perform double-blind placebo-controlled studies on the effect of different doses of epinephrine used during anaphylaxis. Recently, Moss et al. [10] proposed that a method to further investigate optimal dosing could involve documentation of serum epinephrine levels in patients reacting during oral provocation challenges. However, patients react to different degrees with different symptoms to different amounts of ingested or injected allergen and there is also variability in the time from exposure to reaction. Thus, we do not believe it to be feasible to design and evaluate studies on comparing doses of epinephrine in clinical trials/practice by this method either.

### Recommended doses lower than 0.01 mg epinephrine/kg body weight

Doses lower than the arbitrarily selected dose of 0.01 mg/kg body weight are given when children/adolescents weighing > 15 kg are treated with 0.15 mg epinephrine, patients weighing > 30 kg are treated with 0.3 mg epinephrine, and subjects weighing > 50 kg and treated with 0.5 mg epinephrine, as delineated in Table 1. Thus in most cases, when patients are prescribed auto-injectors, the majority are prescribed a dose lower than 0.01 mg/kg (Tables 1, 2, 3, and Figs. 1 and 2). For example, a 0.5 mg dose would result in dosing of 0.003 mg/kg in patients weighing 150 kg and a 0.3 mg dose would result in 0.002 mg/kg epinephrine. These doses would result in approximately one-third and one-fifth of the recommended dose, respectively. The use of lower dosing has presumably been due to fear of ADRs to epinephrine, particularly in adults, as well as the varying availability of each auto-injector dose in countries around the globe.

In addition to dosing concerns, obese or overweight patients typically have skin to muscle distance that is longer than the length of the needle of the auto-injector, which may result in subcutaneous injection [11] and slower systemic distribution than intramuscular (IM) injection [12, 13]. Both inadequate needle length and the use of the lower-dose 0.3 mg auto-injector may provide some explanation for the relative increase in anaphylaxis fatalities in women with higher body mass indices in the community; a well-documented phenomenon [8, 14–16].

### Recommended doses higher than 0.01 mg/kg body weight

Often, children have received higher doses than those internationally agreed upon when prescribed epinephrine auto-injectors. This has occurred when children weighing less than 15 kg receive auto-injectors containing 0.15 mg, children weighing < 30 kg receive 0.3 mg, and those weighing < 50 kg are prescribed 0.5 mg (Table 2).

The highest doses per kg body weight are prescribed to children weighing less than 15 kg (Table 1). In many countries, auto-injector prescriptions in this population is still in keeping with national dose recommendations, assuming an allergist finds epinephrine to be indicated (Table 2). Even the EAACI indicates that 0.15 mg can be prescribed to children weighing 7.5 kg or more; despite the fact that in a 7.5 kg child, this would lead to double the generally recommended mg/kg dose. The AAAAI/ACAAI do not suggest any lower weight-limit for the 0.15 mg dose [17], whereas the Canadian Society of Allergy and Clinical Immunology recommends 0.15 mg for all children less than 15 kg with the caveat that this should be revisited when a lower dose becomes available [18].

There is one lower-dose auto-injector, Auvi-Q^®^ 0.1 mg, available in the United States and officially indicated for infants weighing 7.5 to 15 kg. Even when using the Auvi-Q^®^ 0.1 mg device, the dose delivered is higher than the generally recommended in all children weighing less than 10 kg.

### Recommended dosing according to age

The Resuscitation Council of the UK [8] recommends dosing according to age, using 6 months of age as the limit between 0.1 and 0.15 mg, 6 years as the limit between 0.15 and 0.3 mg, and 12 years as the limit between 0.3 and 0.5 mg. These limits are approximations of the weight-based limits generally agreed upon 10, 15, 30, and 50 kg [8]. They propose using 0.3 mg for thin and or prepubertal adolescents older than 12 years. This principle has been adopted by the Portuguese Society of Allergy and Clinical Immunology, and by the South African society (Table 3). Using somewhat of a hybrid approach, The Hungarian Society recommends following the weight recommendations by EAACI based on weight but with an increase from 0.15 to 0.3 mg at 14 years of age (Table 3).

Kim et al. performed studies on young children to adults assessing the skin to muscle distance in food allergic subjects [19]. Using the data from these studies, the weights of the allergic children 6 to 12 years of age receiving 0.3 mg epinephrine, would compare to 0.020–0.004 mg/kg body weight; or double-to-half the generally accepted dose per kilogram [19]. Similarly, the dose of 0.5 mg recommended for children 12–18 years of age would result in an epinephrine dose varying between 0.0160 and 0.006 mg/kg, and in overweight or obese adults only 0.003 mg/kg body weight.

### The lethal dose of epinephrine

The lethal dose of epinephrine has not been investigated in humans prospectively for obvious reasons. With this is mind, there are at least two potential ways one may study possible adverse reactions to epinephrine resulting in death; accidental overdose data in humans and testing the Lethal Dose of 50% (LD_50_) in animals.

There are data from studies in mice indicating that the LD_50_ is about 3 mg/kg body weight [20]. Consequently, the LD_50_ for epinephrine in mice is much higher than the dose used for the treatment of systemic allergic reactions/anaphylaxis in the community (Table 4). Therefore, one could conclude that based on animal studies, the recommended doses used in humans should be safe and tolerated by patients without cardiac disease.

**Table 4:**
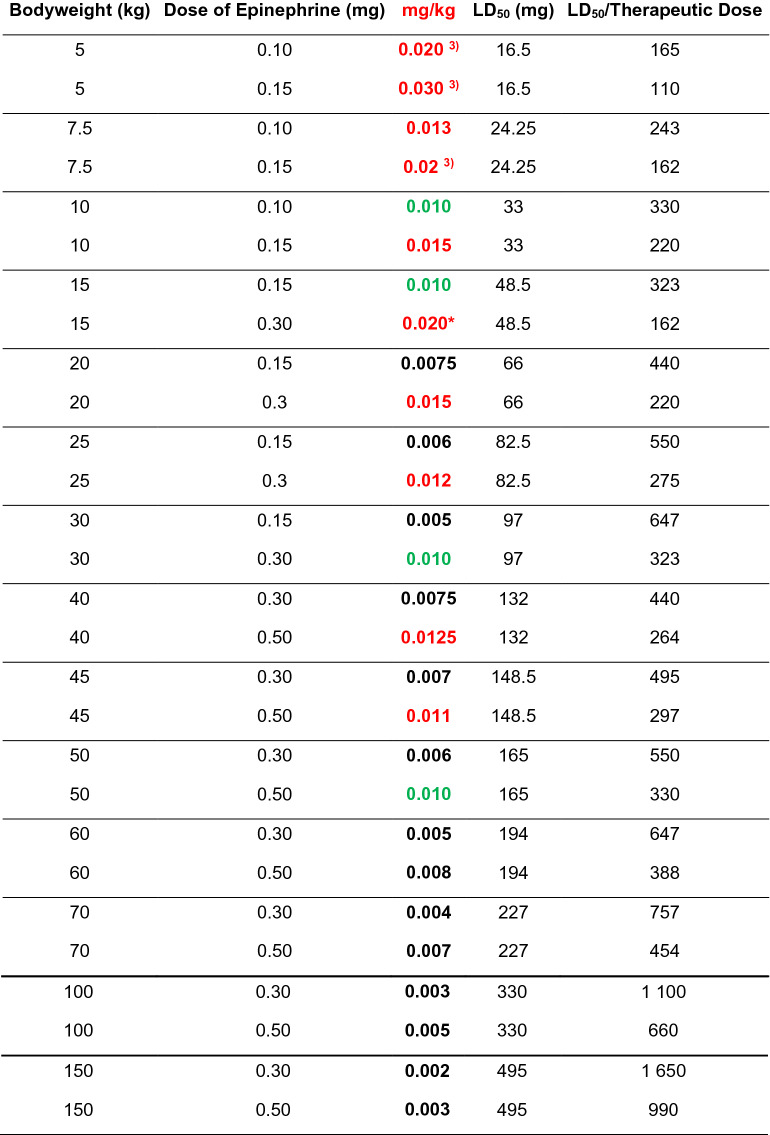
Comparison of doses used in humans compared to LD_50_ when tested in a mouse model

Colour code: numbers in red denote doses higher than the “recommended dose” 0.01 mg/kg body weight, green color indicates “the recommended dose”, 0.01 mg/kg body weight, and black color doses lower than the “recommended” dose. Yellow areas indicate that the dose can only be prescribed in some countries, leading to (probably) under-medication

LD_50_ in mice was 3.3 mg per kg body weight (confidence intervals 2.1–4.6 mg/kg body weight) [20]. Two different doses are given for each weight. (1) The 0.1 mg dose is only available in the US. (2) The 0.5 mg dose is available in some European countries (Germany, UK, Ireland, The Netherlands, France, Spain, Italy, Czech Republic, Finland, Denmark, Sweden, Norway) and Canada

*mg* milligrams, *kg* kilograms

There are few publications on high-dose epinephrine administration in patients experiencing anaphylaxis. Clinically, medication errors due to confusion of cardiac arrest dosing with anaphylaxis dosing of epinephrine may be responsible. During cardiac arrest, treatment with a high dose push of 1 mg of epinephrine [1:10,000 weight/volume (w/v)] intravenously (IV) is recommended; a dose much higher than that used in treatment of anaphylaxis.

To confuse things further, recommendations for treatment of anaphylaxis resistant to IM injection of epinephrine include the establishment of a slow IV infusion with 0.1 mg epinephrine at a concentration of 1:10,000 w/v. These overlapping dosages and concentrations have also led to several medication errors and medical complications [21]. In some cases, patients with systemic allergic reactions/anaphylaxis have received an IV push dose of 1 mg epinephrine, resulting in severe adverse reactions [21, 22]]. Side effects have included immediate loss of consciousness, hypotension, tachypnoea, hypoxemia despite O2-supplementation [22], and even transient ST-elevations on ECG [21].

There have also been deaths reported after topical application of epinephrine 1 mg/mL (1:1000 w/v) onto a wound site, after injection of 3 mg of 1:1000 w/v epinephrine into the ear [23]. Lindor et al. also reported on “inappropriate IV epinephrine dosing”; i.e. IV injection of very high doses of epinephrine in 5 cases [24].

The influence of epinephrine on patients with easily triggered cardiac complications such as arrhythmias and a tendency towards myocardial infarction must be considered whenever injecting this medication.

## Practical clinical implications

### Is there a need for adjustment of international dose recommendations?

**Fig. 3:**
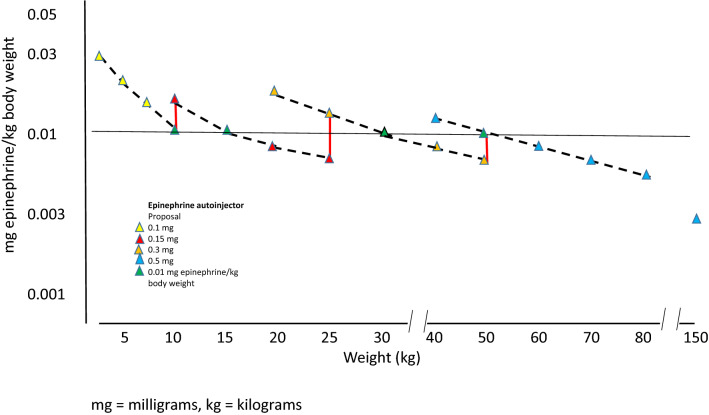
Proposed improvement of effective epinephrine delivery per kilogram body weight by our recommendations

In children, a higher dose than that generally recommended appears to be well tolerated. Therefore, it is likely reasonable to at least increase the dose to 0.15 mg in children weighing 10–25 kg and to 0.3 mg in children weighing 25–40 kg. Likewise, the 0.5 mg dosing should be tolerated by adolescents and young adults weighing more than 40 kg. Healthy adults weighing more than 50 kg would likely benefit from the 0.5 mg dose [11] (Table 5 and Fig. 3).

**Table 5:**
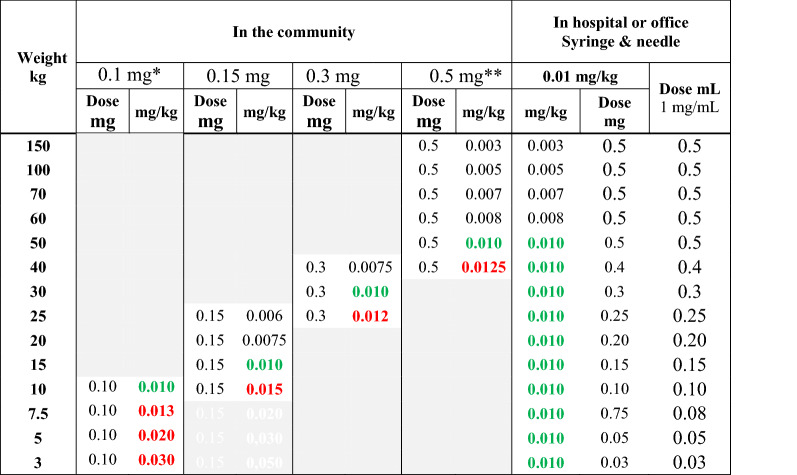
Proposed increased dosing of adrenaline by auto-injectors

Dosing of epinephrine using the 0.1, 0.15, 0.3 and 0.5 mg doses in children and adults. The 0.01 mg/kg dose given by health care personnel in health care settings. Color-codes as in Table 1

^*^Available only in USA

^**^Available only in some European countries (Germany, UK, Ireland, The Netherlands, France, Spain, Italy, Czech Republic, Finland, Denmark, Sweden, Norway) and Canada

### Is there a need for a wider variety of available doses of epinephrine?

From the data presented, it seems reasonable to introduce an auto-injector containing 0.1 mg epinephrine to other countries in addition to the US. Furthermore, it would be valuable to make auto-injectors containing 0.5 mg epinephrine available in all countries. This dose has been used for the treatment of anaphylaxis in adults and teens clinically for decades, and has been used extensively for the treatment of anaphylaxis in Western Europe and Canada without any major complications reported. While it is unlikely feasible to fully customize an epinephrine auto-injector to each individual patient weight and skin-to-muscle distance, there is evidently a need for dose optimization for many patients.

### Discussion

The doses of epinephrine recommended by international and national societies are in many cases conservative and vary between countries of the same region. Discussion surrounding epinephrine doses delivered by auto-injectors is critical, as exemplified by the cases of women who died after severe systemic allergic reactions in the community despite them being treated with two timely doses of epinephrine 0.3 mg. In these cases, aside from inadequate dosing, the inadequate needle lengths of auto-injectors have been implicated [15, 25]. In at least one case in the UK [15], the confirmed depth of auto-injector delivery was shorter (16 mm) than the distance from the skin to the muscle (22 mm), and thus the injection was probably delivered subcutaneously.

We believe that the conservative dosing of epinephrine auto-injectors recommended by international and national societies may contribute to the deaths from anaphylaxis in some patients. Therefore, in patients who are otherwise healthy, we propose that care providers preferentially prescribe auto-injectors containing 0.15 mg epinephrine for children weighing 10 to 25 kg, and auto-injectors containing 0.3 mg epinephrine for those weighing 25 to 40 kg. Since auto-injectors labelled 0.15 mg have been used routinely in many children weighing less than 10 kg, we do not believe it to be mandatory to recommend use of 0.1 mg auto-injectors for children weighing ≤ 10 kg given their limited availability outside the US at this time.

Furthermore, we propose the use of auto-injectors labelled 0.5 mg for those weighing more than 40 kg, since such auto-injectors induce a higher maximum serum concentration (C_max_) than auto-injectors containing 0.3 mg epinephrine [8, 12] and have been used extensively in both Europe and Canada without reported adverse reactions [8].

## Data Availability

Due to the nature of this review article, the datasets generated and/or analyzed are publicly available at the reference addresses listed.

## References

[1] Stolz F (1904). Über adrenalin und alkylaminoacetobrenzcatechin. Ber Dtsch Chem Ges.

[2] Dakin HD (1905). The synthesis of a substance allied to adrenaline. Proc R Soc Lond.

[3] Lockey SD (1980). A new method of administering aqueous epinephrine: the EpiPen, an automatic syringe. J Asthma Res.

[4] Jayamali WD, Herath H, Kulathunga A (2017). Myocardial infarction during anaphylaxis in a young healthy male with normal coronary arteries- is epinephrine the culprit?. BMC Cardiovasc Disord.

[5] Ruiz-Garcia M, Bartra J, Alvarez O, Lakhani A, Patel S, Tang A (2020). Cardiovascular changes during peanut-induced allergic reactions in human subjects. J Allergy Clin Immunol.

[6] Turner PJ, Ruiz-Garcia M, Durham SR, Boyle RJ (2020). Limited effect of intramuscular epinephrine on cardiovascular parameters during peanut-induced anaphylaxis: an observational cohort study. J Allergy Clin Immunol Pract.

[7] Grabenhenrich LB, Dolle S, Rueff F, Renaudin JM, Scherer K, Pfohler C (2018). Epinephrine in severe allergic reactions: the European Anaphylaxis Register. J Allergy Clin Immunol Pract.

[8] Dodd A, Hughes A, Sargant N, Whyte AF, Soar J, Turner PJ (2021). Evidence update for the treatment of anaphylaxis. Resuscitation.

[9] Correa N, Quidwai A, Jeimy S, Rondilla N, White F, Moote W (2021). Multicenter real-world experience with epinephrine 0.5 mg dosing for anaphylaxis with allergen immunotherapy. Immunotherapy.

[10] Moss J, Jani Y, Edwards B, Tomlin S, Rashed AN (2020). Pharmacokinetic and pharmacodynamic evidence of adrenaline administered via auto-injector for anaphylactic reactions: a review of literature. Br J Clin Pharmacol.

[11] Dreborg S, Tsai G, Kim H (2019). Epinephrine autoinjector needles. Does height and BMI add valuable information in adults. Glob J Immunol Allergic Dis.

[12] Dreborg S, Kim H (2021). The pharmacokinetics of epinephrine/adrenaline autoinjectors. Allergy Asthma Clin Immunol: Off J Can Soc Allergy Clin Immunol.

[13] Duvauchelle T, Robert P, Donazzolo Y, Loyau S, Orlandini B, Lehert P (2018). Bioavailability and cardiovascular effects of adrenaline administered by anapen autoinjector in healthy volunteers. J Allergy Clin Immunol Pract.

[14] Pumphrey RS (2000). Lessons for management of anaphylaxis from a study of fatal reactions. Clin Exp Allergy: J Br Soc Allergy Clin Immunol.

[15] Pumphrey RSH. Autopsy of Poppy Harvey. 2014.

[16] Inquiring into the death touching Poppy Harway, Summing up: hearing before the HM Coroner’s Court, Ipswich, H.M. Coroner’s Court, Ipswich (June 28th, 2011, 2011).

[17] Lieberman P, Nicklas RA, Oppenheimer J, Kemp SF, Lang DM, Bernstein DI (2010). The diagnosis and management of anaphylaxis practice parameter: 2010 update. J Allergy Clin Immunol.

[18] Halbrich M, Mack DP, Carr S, Watson W, Kim H (2015). CSACI position statement: epinephrine auto-injectors and children < 15 kg. Allergy Asthma Clin Immunol: Off J Can Soc Allergy Clin Immunol.

[19] Kim L, Nevis IF, Tsai G, Dominic A, Potts R, Chiu J (2014). Children under 15 kg with food allergy may be at risk of having epinephrine auto-injectors administered into bone. Allergy Asthma Clin Immunol: Off J Can Soc Allergy Clin Immunol.

[20] Volpato MC, Ranali J, Amaral IMG, Demetrio CMG, Chalita LVAS (1999). Acute toxicity (LD50 and CD50) of lidocain and pilocaine in combination with adrenaline and felypressin. Indian J Dent Res.

[21] Kanwar M, Irvin CB, Frank JJ, Weber K, Rosman H (2010). Confusion about epinephrine dosing leading to iatrogenic overdose: a life-threatening problem with a potential solution. Ann Emerg Med.

[22] Andre MC, Hammer J (2019). Life-threatening accidental intravenous epinephrine overdose in a 12-year-old boy. Pediatr Emerg Care.

[23] Lindor RA, McMahon EM, Wood JP, Sadosty AT, Boie ET, Campbell RL (2018). Anaphylaxis-related malpractice lawsuits. West J Emerg Med.

[24] Grissinger M (2013). Fatalities after inadvertent injections of topical epinephrine. P T.

[25] Pumphrey RS, Roberts IS (2000). Postmortem findings after fatal anaphylactic reactions. J Clin Pathol.

[26] Muraro A, Roberts G, Worm M, Bilo MB, Brockow K, Fernandez Rivas M (2014). Anaphylaxis: guidelines from the European Academy of Allergy and Clinical Immunology. Allergy.

[27] Ring J, Beyer K, Biedermann T, Bircher A, Duda D, Fischer J (2014). Guideline for acute therapy and management of anaphylaxis: S2 Guideline of the German Society for Allergology and Clinical Immunology (DGAKI), the Association of German Allergologists (AeDA), the Society of Pediatric Allergy and Environmental Medicine (GPA), the German Academy of Allergology and Environmental Medicine (DAAU), the German Professional Association of Pediatricians (BVKJ), the Austrian Society for Allergology and Immunology (OGAI), the Swiss Society for Allergy and Immunology (SGAI), the German Society of Anaesthesiology and Intensive Care Medicine (DGAI), the German Society of Pharmacology (DGP), the German Society for Psychosomatic Medicine (DGPM), the German Working Group of Anaphylaxis Training and Education (AGATE) and the patient organization German Allergy and Asthma Association (DAAB). Allergo J Int.

[28] Gottberg L. Anafylaxi. Rekommendationer för omhädnertagande och behandling. (Anaphylaxis. Recommendations for care and treatment). Stockholm; 2013.

[29] Ewan PW, Dugue P, Mirakian R, Dixon TA, Harper JN, Nasser SM (2010). BSACI guidelines for the investigation of suspected anaphylaxis during general anaesthesia. Clin Exp Allergy: J Br Soc Allergy Clin Immunol.

